# Distributed State Observer for Systems with Multiple Sensors under Time-Delay Information Exchange

**DOI:** 10.3390/s24134382

**Published:** 2024-07-05

**Authors:** Wen Fang, Fanglai Zhu

**Affiliations:** College of Electronics and Information Engineering, Tongji University, Shanghai 201804, China; fangwen@tongji.edu.cn

**Keywords:** distributed observer, multiple sensors, time delay, linear matrix inequality, Lyapunov stability theory

## Abstract

The issues of state estimations based on distributed observers for linear time-invariant (LTI) systems with multiple sensors are discussed in this paper. We deal with the scenario when the information exchange has known time delays, and aim at designing a distributed observer for each subsystem such that each distributed observer can estimate the system state asymptotically by rejecting the time delay. To begin with, by rewriting the target system in a connecting form, a subsystem which is affected by the time-delay states of other nodes is established. And then, for this subsystem, a distributed observer with time delay is constructed. Moreover, an equivalent state transformation is made for the observer error dynamic system based on the observable canonic decomposition theorem. Further, in order to ensure that the distributed observer error dynamic system is asymptotically stable even if there exists a time delay, a linear matrix inequality (LMI) which is relative to the Laplace matrix is elaborately set up, and a special Lyapunov function candidate based on the LMI is considered. Next, based on the Lyapunov function and Lyapunov stability theory, we prove that the error dynamic system of the distributed observer is asymptotically stable, and the observer gain is determined by a feasible solution of the LMI. Finally, a simulation example is given to illustrate the effectiveness of the proposed method.

## 1. Introduction

A state observer is a dynamic system which is constructed by using the measurement output together with the control input of the original system, such that the dynamic system can generate the state estimates of the original system. The motivation of developing the state estimation concept is that for most practical systems, the state information is difficult, expensive or even impossible to measure directly. The model-based state estimation theory has attracted extensive attention and has been gradually improved to meet the needs of practical engineering applications since the Luenberger observer was proposed in the 1960s [1,2]. After that, all kinds of observer design methods corresponding to various complex scenarios have been developed, such as the unknown input observer (UIO)  [3,4], the time-delay observer [5,6], the sliding model observer [7,8,9], and the reduced-order observer [10,11]. For example, Zhu et al. propose a joint UIO which is able to offer the asymptotic convergent estimations of state and unknown input simultaneously [3]. Edwards et al. design a sliding model observer to reconstruct the actuator fault [7]. In [11], a reduced-order observer is constructed for a descriptor system with disturbance appearing in both the state equation and output equation.

Recently, the distributed observer which can reconstruct the entire system states based on partial output information through information exchange has gained much attention. For a target system monitored by a group of sensor nodes, the objective of a distributed observer is to asymptotically estimate the state of the target system using its local measurements together with information exchange with neighbors. The motivations for designing distributed observers are mainly of two kinds. To begin with, it is particularly beneficial for large-scale systems, such as the electrical power system [12,13], water irrigation system [14], intelligent vehicles [15] and so on, to obtain state estimates using multiple sensors deployed in different geographic or spatial locations. Sensors with computation and communication functions are available and this allows integration between observers and local sensors. To date, many significant results on distributed observers have been published in the literature [12,13,14,15,16,17,18,19,20,21,22,23,24,25,26,27,28]. For example, a theoretically robust and computationally efficient distributed state estimator is proposed in [15], which is a typical application of a distributed observer in a power system. By using the observability decomposition of target systems and introducing an auxiliary undirected graph, paper [16] discusses a Luenberger-type distributed observer for linear systems based on LMIs and then a Luenberger-type distributed observer design method is given. An approach for solving the problem of the distributed state estimation of LTI systems is proposed in [18], where the distributed state observation task is reformulated as a parameter estimation problem, and the estimation convergence is achieved in finite time.

In the domain of networked control systems, many challenging problems require solution. For example, Cai et al. [19] develop a novel control scheme that ensures mean square leader–follower consensus in the presence of unknown transfer probabilities and system perturbations. The proposed method incorporates an observer to reconstruct system states and an adaptive event-triggered mechanism to dynamically adjust communication, thereby enhancing the overall efficiency and robustness of the MASs. Cao et al. [20] present an adaptive NN-based observer for MASs with time-varying delays, offering a low-gain approach to enhance the state estimating and tracking accuracy through a dynamic event-triggered control strategy. In [21], an adaptive NN and low-gain observer in a dynamic event-triggered control framework is introduced to ensure fixed-time cooperative formation for MASs. Wang et al. [22] propose an observer-based sliding mode control approach for networked fuzzy singularly perturbed systems, enhancing system stability and performance under the weighted try-once-discard protocol. Different from traditional observers, distributed observers have to be dealt with in terms of the network construction. As a result, one of the exclusive features of distributed observers is that information exchange is necessary such that each local observer is able to generate the state estimates. It is this exclusive feature that implies there are extra concerns when we design a distributed observer, especially with respect to requirements from information exchange links. These problems include data transfer delays, data transfer blocks, data losses, or even communication channels suffering from malicious attacks. For distributed observer designs, coping with the above-mentioned problems has been taken into account by some researchers [23,24,25,26,27,28,29,30,31,32]. For example, ref. [23] provides a survey of recent advances in distributed event-triggered estimation for dynamical systems operating over resource-constrained sensor networks. In paper [24], a hybrid observer is discussed which can provide estimations asymptotically even if one or several agents join or leave the internet. Aiming at the multi-observer network system with time delay and packet loss in information communication, paper [25] proposes a distributed estimation problem of system state realized by a continuous time-distributed observer. In paper [26], the distributed estimation problem is solved based on LMI, where the digital communication between the observer nodes is modeled by the time-delay approach in which variable sampling intervals, transmission delays, and packet dropouts are taken into account. Papers [27,28] focus on the construction of distributed observers in the presence of arbitrarily large communication time delays. Paper [29] designs a distributed observer for scenarios of both state-dependent and non-state-dependent noise occurring in information exchange. The issues of the communication delay and communication frequency in distributed state estimation using a unified structure are addressed in [30].

Based on the above observations, in this paper, we focus on coping with the problems of information communication delays in constructing a distributed observer for an LTI system. The major contributions of the paper are summarized as follows:

(1) By making good use of the special structure of the Laplacian matrix of the communication topology, the state equation of the target system is rewritten in a connecting form, while the information transfer delay is considered. In this way, a distributed observer design model with information communication delays is set up.
(2) Referring to the design model, a distributed observer is designed, in which the time delay caused by the information communication is robustly rejected by constructing a special Lyapunov function which contains two parts, which are dependent on each other through an LMI which is predefined elaborately. And the observer gains can be obtained by solving a single LMI.

The rest of the paper is organized as follows: In Section 2, notations, concepts used, and a system description are given. In Section 3, the main results about the distributed observer which can reject the time delay are presented. In Section 4, a simulation example is given to illustrate the effectiveness of the proposed method. Section 5 gives the conclusions.

## 2. Preliminaries and System Description

### 2.1. Notation

**Notation 1.**  *For a square symmetric matrix $M\in R^{n\times n}$, M<0 (M>0) means that M is a symmetric negative (positive) definite matrix. Suppose $G_{i}$ ($i\in N=\left\{1,\cdots ,N\right\}$) are N matrices or scalars, notation $\underset{i\in N}{\mathrm{diag}}\left(G_{i}\right)$ stands for a diagonal (block) matrix with $G_{i}$ (i=1,⋯,N) being the diagonal entries (block matrices).*

### 2.2. Basic Graph Theory

For a complex network with *N* nodes labeled by 1,⋯,N, the information flow among the N nodes is described by a directed weighted graph $G\left(V,E\right)$, where V=$\left\{v_{1},\cdots ,v_{N}\right\}$ stands for the node set $A=\left[a_{ij}\right]\in R^{N\times N}$, and E=V×V denotes the edge set. Further, define an adjacency matrix to describe the connection condition between any two nodes. Specifically, set $a_{ij}=1$ if $\left(v_{i},v_{j}\right)\in E$, which means that node i can receive information from node j; and $a_{ij}=0$ if $\left(v_{i},v_{j}\right)\notin E$. We always assume that $\left(v_{i},v_{i}\right)\notin E$, which implies that $a_{ij}=0$ for all i∈N. Let $L=\left[l_{ij}\right]\in R^{N\times N}$ be the Laplacian matrix with $l_{ij}=-a_{ij}\left(i\neq j\right)$ and $l_{ii}=\sum_{k=1}^{N}a_{ik}$. If we further define $B=\underset{i\in N}{\mathrm{diag}}\left(\sum_{j=1}^{N}a_{ij}\right)$, then we have L=B−A.

**Definition 1.**  *The graph G is said to be strong connected if there exists a directed path for any two notes in G.*

**Lemma 1**  ([31])**.** *Let G be a strongly connected directed graph with N nodes and L be its Laplacian matrix. Then, the following statements hold.*

*(i) There exists a vector $\theta ={\left[\begin{array}{ccc} \theta_{1} & \cdots & \theta_{N} \end{array}\right]}^{T}$ satisfying $0<\theta_{i}<1$, $\sum_{i=1}^{N}\theta_{i}=1$ and $\theta^{T}L=0$.*

*(ii) Define matrix $\hat{L}=\Theta L+L^{T}\Theta$ with $\Theta =\underset{i\in N}{\mathrm{diag}}\left(\theta_{i}\right)$, then $\hat{L}$ is a symmetric Laplacian matrix associated with a connected undirected graph obtained from G by ignoring the directions of all the edges.*

### 2.3. System Formulation

Consider a linear system with *N* sensors, where the ith node or subsystem is described by

(1) $$\left\{\begin{array}{c} \dot{x}\left(t\right)=Ax\left(t\right)+Bu\left(t\right)\\ y_{i}\left(t\right)=C_{i}x\left(t\right) \end{array},\;i\in N\right.$$

where $x\left(t\right)\in R^{n}$, $y\left(t\right)\in R^{p}$ and $u\left(t\right)\in R^{m}$ are the system state, measurement output and control input vectors, respectively. $A\in R^{n\times n}$, $B\in R^{n\times m}$ and $C_{i}\in R^{p_{i}\times n}\left(i\in N\right)$ are all known constant matrices. Here, we assume that $\left(A,C_{i}\right)$ is neither observable nor detectable for all i∈N. Therefore, for the ith subsystem, it is impossible for one to design an observer to obtain the state estimation of the system only by using the local sensor output $y_{i}$. Moreover, we assume that the pair $\left(A,C\right)$ is observable, where $C={\left[\begin{array}{ccc} C_{1}^{T} & \cdots & C_{N}^{T} \end{array}\right]}^{T}\in R^{p}$, and $p=p_{1}+\cdots +p_{N}$.

## 3. Distributed Observer with Communication Time Delay

From the construction of the Laplacian matrix $L=\left[l_{ij}\right]\in R^{N\times N}$, we know that $\sum_{j=1}^{N}l_{ij}=0\left(i\in N\right)$. Thus, the ith subsystem (1) can also be rewritten as

(2) $$\dot{x}\left(t\right)=\left(A+K_{i}C_{i}\right)x\left(t\right)-cT_{iu}^{T}T_{iu}\sum_{j=1}^{N}l_{ij}x\left(t-\tau \right)-K_{i}y_{i}\left(t\right)+Bu\left(t\right)$$

where c>0 is a scalar and $K_{i}\in R^{n\times p_{i}}$ is a gain matrix to be designed later, while τ>0 is an arbitrary scalar which stands for the time delay due to the information exchange between any two subsystem and it is assumed to be known.

Since $\left(A,C_{i}\right)$ is unobservable, which means that $\mathrm{rank}\left(O_{i}\right)=v_{i}<n$, where $O_{i}={\left[\begin{array}{cccc} C_{i}^{T} & {\left(C_{i}A\right)}^{T} & \cdots & {\left(C_{i}A^{n-1}\right)}^{T} \end{array}\right]}^{T}$. Thus, based on the observable canonic decomposition theorem, there must exist an orthogonal matrix $T_{i}\in R^{n\times n}$ satisfying $T_{i}T_{i}^{T}=T_{i}^{T}T_{i}=I_{n}$ such that $T_{i}AT_{i}^{T}=\left[\begin{array}{cc} A_{id} & 0\\ A_{ir} & A_{iu} \end{array}\right]$ and $C_{i}T_{i}^{T}=\left[\begin{array}{cc} C_{id} & 0 \end{array}\right]$ with $A_{id}\in R^{v_{i}\times v_{i}}$, $A_{ir}\in R^{\left(n-v_{i}\right)\times v_{i}}$, $A_{iu}\in R^{\left(n-v_{i}\right)\times \left(n-v_{i}\right)}$ and $C_{id}\in R^{p_{i}\times v_{i}}$. Further, the pair $\left(A_{id},C_{id}\right)$ is observable. For the discussion, we need to decompose matrix $T_{i}$ into a black matrix as $T_{i}={\left[\begin{array}{cc} T_{id}^{T} & T_{iu}^{T} \end{array}\right]}^{T}$, where $T_{id}\in R^{v_{i}\times n}$ and $T_{iu}\in R^{(n-v_{i})\times n}$.

**Remark 1.**  *To design a distributed observer, we rewrite system (1) into (2), by making good use of the property of the information communication topology, such that the rewritten system (2) is in the form of a distributed feature. Moreover, in this way, the time delay, which is caused because of the information exchange, is characterized in the model. Therefore, based on the rewritten system (2), we can conveniently handle the time delay problem brought by the information communication.*

Now, design a distributed observer for system (2) as follows:

(3) $${\dot{\hat{x}}}_{i}\left(t\right)=\left(A+K_{i}C_{i}\right)\hat{x}\left(t\right)-cT_{iu}^{T}T_{iu}\sum_{j=1}^{N}l_{ij}{\hat{x}}_{j}\left(t-\tau \right)-K_{i}y_{i}\left(t\right)+Bu\left(t\right)$$

The observer error dynamic system can be obtained by subtracting (3) from (2):

(4) $${\dot{\tilde{x}}}_{i}\left(t\right)=\left(A+K_{i}C_{i}\right)\tilde{x}\left(t\right)-cT_{iu}^{T}T_{iu}\sum_{j=1}^{N}l_{ij}{\tilde{x}}_{j}\left(t-\tau \right)$$

where ${\tilde{x}}_{i}\left(t\right)=x\left(t\right)-{\hat{x}}_{i}\left(t\right)$. In what follows, make an equivalent state transformation of ${\tilde{\zeta }}_{i}=T_{i}{\tilde{x}}_{i}$(i∈N), and choose $K_{i}=T_{i}^{T}\left[\begin{array}{c} K_{id}\\ 0_{\left(n-v_{i}\right)\times p_{i}} \end{array}\right]\in R^{n\times p_{i}}$ with $K_{id}\in R^{v_{i}\times p_{i}}$ being a gain matrix to be determined later, then we obtain an equivalent system of (4) as follows:

(5) $${\dot{\tilde{\zeta }}}_{i}\left(t\right)={\bar{A}}_{i}{\tilde{\zeta }}_{i}\left(t\right)-cF_{i}\sum_{j=1}^{N}l_{ij}T_{j}^{T}{\tilde{\zeta }}_{j}\left(t-\tau \right)$$

where ${\bar{A}}_{i}=\left[\begin{array}{cc} A_{id}+K_{id}C_{id} & 0\\ A_{ir} & A_{iu} \end{array}\right]$ and $F_{i}=T_{i}T_{iu}^{T}T_{iu}=\left[\begin{array}{c} 0_{v_{i}\times \left(n-v_{i}\right)}\\ T_{iu} \end{array}\right]$.

Moreover, denote $\zeta_{i}={\left[\begin{array}{cc} \zeta_{id}^{T} & \zeta_{iu}^{T} \end{array}\right]}^{T}$, ${\hat{\zeta }}_{i}={\left[\begin{array}{cc} {\hat{\zeta }}_{id}^{T} & {\hat{\zeta }}_{iu}^{T} \end{array}\right]}^{T}$, ${\tilde{\zeta }}_{id}=\zeta_{id}$$-{\hat{\zeta }}_{id}$ and ${\tilde{\zeta }}_{iu}=\zeta_{iu}-{\hat{\zeta }}_{iu}$ with $\zeta_{id},{\hat{\zeta }}_{id},{\tilde{\zeta }}_{id}\in R^{v_{i}}$ and $\zeta_{iu},{\hat{\zeta }}_{iu},{\tilde{\zeta }}_{iu}$$\in R^{n-v_{i}}$, then, (5) can also be rewritten as

(6) $$\left\{\begin{array}{c} {\dot{\tilde{\zeta }}}_{id}\left(t\right)=\left(A_{id}+K_{id}C_{id}\right){\tilde{\zeta }}_{id}\left(t\right)\\ {\dot{\tilde{\zeta }}}_{iu}\left(t\right)=A_{ir}{\tilde{\zeta }}_{id}\left(t\right)+A_{iu}{\tilde{\zeta }}_{iu}\left(t\right)-cT_{iu}\sum_{j=1}^{N}l_{ij}T_{jd}^{T}{\tilde{\zeta }}_{jd}\left(t-\tau \right)-cT_{iu}\sum_{j=1}^{N}l_{ij}T_{ju}^{T}{\tilde{\zeta }}_{ju}\left(t-\tau \right) \end{array}\right.$$

The overall system of the first equation of (6) is

(7) $${\dot{\tilde{\zeta }}}_{d}\left(t\right)=\left(A_{d}+K_{d}C_{d}\right){\tilde{\zeta }}_{d}\left(t\right)$$

where ${\tilde{\zeta }}_{d}={\left[\begin{array}{ccc} {\tilde{\zeta }}_{1d}^{T} & \cdots & {\tilde{\zeta }}_{Nd}^{T} \end{array}\right]}^{T}$, $A_{d}=\underset{i\in N}{\mathrm{diag}}\left(A_{id}\right)\in R^{v\times v}$, $K_{d}=\underset{i\in N}{\mathrm{diag}}\left(K_{id}\right)\in R^{v\times p}$ and $C_{d}=$$\underset{i\in N}{\mathrm{diag}}\left(C_{id}\right)\in R^{p\times v}$. The overall system of the second equation of (6) is

(8) $${\dot{\tilde{\zeta }}}_{u}\left(t\right)=A_{r}{\tilde{\zeta }}_{d}\left(t\right)+A_{u}{\tilde{\zeta }}_{u}\left(t\right)-cT_{u}\left(L⊗I_{n}\right)T_{d}^{T}{\tilde{\zeta }}_{d}\left(t-\tau \right)-cT_{u}\left(L⊗I_{n}\right)T_{u}^{T}{\tilde{\zeta }}_{u}\left(t-\tau \right)$$

where ${\tilde{\zeta }}_{u}={\left[\begin{array}{ccc} {\tilde{\zeta }}_{1u}^{T} & \cdots & {\tilde{\zeta }}_{Nu}^{T} \end{array}\right]}^{T}$, $A_{u}=\underset{i\in N}{\mathrm{diag}}\left(A_{iu}\right)\in R^{\left(nN-v)\times (nN-v\right)}$, $A_{r}=\underset{i\in N}{\mathrm{diag}}\left(A_{ir}\right)\in$$R^{(nN-v)\times v}$, $T_{u}=\underset{i\in N}{\mathrm{diag}}\left(T_{iu}\right)\in R^{(nN-v)\times nN}$ and $T_{d}=\underset{i\in N}{\mathrm{diag}}\left(T_{id}\right)\in R^{v\times nN}$. Then, the combination of (7) and (8) is

(9) $${\dot{\tilde{\zeta }}}_{du}\left(t\right)=A_{du}{\tilde{\zeta }}_{du}\left(t\right)-cG{\tilde{\zeta }}_{du}\left(t-\tau \right)$$

where $A_{du}=\left[\begin{array}{cc} A_{d}+K_{d}C_{d} & 0\\ A_{r} & A_{u} \end{array}\right]$, $G=\left[\begin{array}{cc} 0 & 0\\ T_{u}\left(L⊗I_{n}\right)T_{d}^{T} & T_{u}\left(L⊗I_{n}\right)T_{u}^{T} \end{array}\right]$ and ${\tilde{\zeta }}_{du}=$${\left[\begin{array}{cc} {\tilde{\zeta }}_{d}^{T} & {\tilde{\zeta }}_{u}^{T} \end{array}\right]}^{T}$.

**Lemma 2**  ([32])**.** *Under the assumption that this is strongly connected and the pair $\left(A,C\right)$ is detectable, then, $T_{u}\left(\hat{L}⊗I_{n}\right)T_{u}^{T}$ is a symmetric positive definite matrix, where $\hat{L}$ is defined in Lemma 1.*

Suppose that $P_{io}\in R^{v_{i}\times v_{i}}$, $P_{iu}\in R^{\left(n-v_{i}\right)\times \left(n-v_{i}\right)}$, $Q_{io}\in R^{v_{i}\times v_{i}}$ and $Q_{iu}\in R^{\left(n-v_{i}\right)\times \left(n-v_{i}\right)}$ (i=1,⋯,N) are a series of symmetric positive definite matrices, then define $P_{o}=\underset{i\in N}{\mathrm{diag}}\left(P_{io}\right)$, $P_{u}=\underset{i\in N}{\mathrm{diag}}\left(P_{iu}\right)$, $Q_{o}=\underset{i\in N}{\mathrm{diag}}\left(Q_{io}\right)$ and $Q_{u}=\underset{i\in N}{\mathrm{diag}}\left(Q_{iu}\right)$. Moreover, for a proper c>0, construct

(10) $$\Omega_{u}=\gamma Q_{u}-\left[cP_{u}T_{u}\left(\Theta L⊗I_{n}\right)T_{u}^{T}+cT_{u}\left(L^{T}\Theta ⊗I_{n}\right)T_{u}^{T}P_{u}\right]$$

where γ>0 is a scalar. Under the assumption that the LMI

(11) $$cP_{u}T_{u}\left(\Theta L⊗I_{n}\right)T_{u}^{T}+cT_{u}\left(L^{T}\Theta ⊗I_{n}\right)T_{u}^{T}P_{u}<\gamma Q_{u}$$

is feasible for $P_{u}$ and $Q_{u}$, then $P=\left[\begin{array}{cc} P_{o} & \;\\ \; & P_{u} \end{array}\right]$ and $\Omega =\left[\begin{array}{cc} Q_{o} & \;\\ \; & \Omega_{u} \end{array}\right]$ are both symmetric positive definite matrices. Now, for the overall error dynamic system (9), consider the Lyapunov function candidate

(12) $$V\left(t\right)={\tilde{\zeta }}_{du}^{T}\left(t\right)P{\tilde{\zeta }}_{du}\left(t\right)+\int_{t-\tau }^{t}{\tilde{\zeta }}_{du}^{T}\left(s\right)\Omega {\tilde{\zeta }}_{du}\left(s\right)ds$$

The derivative of $V\left(t\right)$ given by (12) along the trajectory of (9) is

(13) $$\dot{V}\left(t\right)=\left[\begin{array}{cc} {\tilde{\zeta }}_{du}^{T}\left(t\right) & {\tilde{\zeta }}_{du}^{T}\left(t-\tau \right) \end{array}\right]\left[\begin{array}{cc} PA_{du}+A_{du}^{T}P+\Omega & -cPG\\ {}* & -\Omega \end{array}\right]\left[\begin{array}{c} {\tilde{\zeta }}_{du}\left(t\right)\\ {\tilde{\zeta }}_{du}\left(t-\tau \right) \end{array}\right]$$

where

(14) $$\begin{array}{c} \left[\begin{array}{cc} PA_{du}+A_{du}^{T}P+\Omega & -cPG\\ {}* & -\Omega \end{array}\right]\\ =\left[\begin{array}{cccc} \Pi_{o} & A_{r}^{T}P_{u} & 0_{v\times v} & 0_{v\times (nN-v)}\\ {}* & \Pi_{u} & -cP_{u}T_{u}\left(L⊗I_{n}\right)T_{d}^{T} & -cP_{u}T_{u}\left(L⊗I_{n}\right)T_{u}^{T}\\ {}* & * & -Q_{o} & 0_{v\times (nN-v)}\\ {}* & * & * & -\Omega_{u} \end{array}\right] \end{array}$$

where $\Omega_{u}$ is determined by (10) and

(15) $$\begin{array}{c} \Pi_{o}=P_{o}A_{d}+A_{d}^{T}P_{o}+X_{d}C_{d}+C_{d}^{T}X_{d}^{T}+Q_{o}\\ \Pi_{u}=P_{u}\left[A_{u}-cT_{u}\left(\Theta L⊗I_{n}\right)T_{u}^{T}\right]+{\left[A_{u}-cT_{u}\left(\Theta L⊗I_{n}\right)T_{u}^{T}\right]}^{T}P_{u}+Q_{u}\\ \Omega_{u}=\gamma Q_{u}-\left[cP_{u}T_{u}\left(\Theta L⊗I_{n}\right)T_{u}^{T}+cT_{u}\left(L^{T}\Theta ⊗I_{n}\right)T_{u}^{T}P_{u}\right] \end{array}$$

with $X_{d}=P_{o}K_{d}$.

**Theorem 1.**  *If the following LMI*

(16) $$\left[\begin{array}{cccc} \Pi_{o} & A_{r}^{T}P_{u} & 0_{v\times v} & 0_{v\times (nN-v)}\\ {}* & \Pi_{u} & -cP_{u}T_{u}\left(L⊗I_{n}\right)T_{d}^{T} & -cP_{u}T_{u}\left(L⊗I_{n}\right)T_{u}^{T}\\ {}* & * & -Q_{o} & 0_{v\times (nN-v)}\\ {}* & * & * & -\Omega_{u} \end{array}\right]<0$$

*has solutions for symmetric positive definite matrices $P_{o}$, $P_{u}$, $Q_{o}$ and $Q_{u}$, then the overall observer time-delay error dynamic system (9) is asymptotically stable.*

**Proof.**  By the Schur complement lemma, LMI (16) is feasible implies that LMI (11) is feasible. Therefore, *P* and Ω are two symmetric positive definite matrices, and this indicates that $V\left(t\right)$ given by (12) is a positive scalar function which can serve as Lyapunov function. It follows from (16) and (14)

(17) $$\left[\begin{array}{cc} PA_{du}+A_{du}^{T}P+\Omega & -cPG\\ {}* & -\Omega \end{array}\right]<0$$

Furthermore, (17) and (13) ensure that the overall error dynamic system (9) is asymptotically stable. Define ${\bar{v}}_{i}=v_{1}+\cdots v_{i};{\bar{p}}_{i}=p_{1}+\cdots +p_{i}\left(i\in N\right)$, ${\bar{v}}_{0}=0$ and ${\bar{p}}_{0}=0$; then, we have the following Algorithm for constructing the distributed time-delay observer (3).    □

**Remark 2.**  *The decentralized control theory is employed to address the distributed estimation problem in [25]. The author provides a general framework for state estimators and outlines constraints on the observer parameters that can influence the convergence rate. Halanay’s inequality is utilized to account for the impact of time-varying delays. In contrast, we have directly proven that the derivative of the Lyapunov functional with respect to time is negative based on selecting a special Lyapunov function candidate which consists of two parts, and the two parts are related with each other. And eventually, the asymptotic stability of the observer error system can be guaranteed by an LMI.*

**Lemma 3.**  *$A_{u}-cT_{u}\left(L⊗I_{n}\right)T_{u}^{T}$ is a Hurwitz matrix provided that c is large enough. And this means that for some $Q_{u}>0$, there exists $P_{u}>0$ such that $\Pi_{u}<0$, where $\Pi_{u}$ is expressed in (15).*

**Proof.**  Define $\Theta =\underset{i\in N}{\mathrm{diag}}\;\left(\theta_{i}\right)$, which is obviously a symmetric positive matrix; then, we have

$${\left(A_{u}-cT_{u}\left(L⊗I_{n}\right)T_{u}^{T}\right)}^{T}\Theta +\Theta \left(A_{u}-cT_{u}\left(L⊗I_{n}\right)T_{u}^{T}\right)=A_{u}^{T}\Theta +\Theta A_{u}-cT_{u}\left(\hat{L}⊗I_{n}\right)T_{u}^{T}$$

where $\hat{L}$ is defined in Lemma 1. By Lemma 2, $T_{u}\left(\hat{L}⊗I_{n}\right)T_{u}^{T}$ is a symmetric positive definite matrix. Therefore, if we choose $c>\frac{2∥A_{u}∥}{\lambda_{\min}\left(T_{u}\left(\hat{L}⊗I_{n}\right)T_{u}^{T}\right)}$, then

$${\left(A_{u}-cT_{u}\left(L⊗I_{n}\right)T_{u}^{T}\right)}^{T}\Theta +\Theta \left(A_{u}-cT_{u}\left(L⊗I_{n}\right)T_{u}^{T}\right)<0$$

As a result, by Lyapunov stability theory, we conclude that $A_{u}-cT_{u}\left(L⊗I_{n}\right)T_{u}^{T}$ is Hurwitz. Furthermore, again by Lyapunov theory, for some $Q_{u}>0$, there exists $P_{u}>0$ such that $\Pi_{u}<0$, where $\Pi_{u}$ is expressed in (15).    □

**Lemma 4.**  *Under the assumption that the following LMI*

(18) $$\left[\begin{array}{cc} \Pi_{u} & P_{u}A_{r}\\ A_{r}^{T}P_{u} & \Pi_{0} \end{array}\right]<0$$

*is feasible, then if there exists a c satisfying*

$$\frac{2∥A_{u}∥}{\lambda_{\min}\left(T_{u}\left(\hat{L}⊗I_{n}\right)T_{u}^{T}\right)}<c<\frac{\lambda_{\min}\left(Y_{1}\right)}{\lambda_{\max}\left(Y_{2}\right)}$$

*then, LMI (16) is feasible, where $Y_{1}=P_{u}A_{r}\Pi_{0}^{-1}A_{r}^{T}P_{u}-\Pi_{u}$ and $Y_{2}=P_{u}T_{u}\left(L⊗I_{n}\right)\cdot$$\left(T_{d}^{T}Q_{0}^{-1}T_{d}\right.+\left.T_{u}^{T}\Omega_{u}^{-1}T_{u}\right)\left(L^{T}⊗I_{n}\right)T_{u}^{T}P_{u}$.*

**Proof.**  LMI (18) is equivalent to $Y_{1}>0$. On the other hand, by the Schur complement lemma, we know that (16) is equivalent to $c^{2}Y_{2}<Y_{1}$, which can be guaranteed by (18).    □

**Remark 3.**  *The feasibility of LMI (16) confirms the feasibility of LMI (11), which can ensure that matrices $P_{u}$ and $\Omega_{u}$ are symmetric positive definite matrices. These two matrices are utilized to construct symmetric positive definite matrices P and* Ω*, and this allows for the selection of Lyapunov function candidate (12) satisfying the condition $V\left(t\right)>0$. Furthermore, the positive definite matrix solution is used to calculate the observer gain matrix $K_{i}$; thereby, the design of our distributed observer can be accomplished. Hence, LMI (16) plays an important role in the designs.*

**Corollary 1.**  *Suppose that the LMI (16) is feasible, and calculate the observer gain matrix $K_{i}$ based on Algorithm 1, then system (3) is a distributed time-delay state observer of the ith subsystem (2) or (1).*

**Algorithm 1** Algorithm for constructing a distributed observer
1:Solve LMI (16) to obtain $P_{o}>0$, $P_{u}>0$, $Q_{o}>0$, $Q_{u}>0$ and $X_{d}$;2:Calculate $K_{d}=P_{o}^{-1}X_{d}$, and then obtain $K_{id}=K_{d}\left(\left({\bar{v}}_{i-1}+1\right):{\bar{v}}_{i},\left({\bar{p}}_{i-1}+1\right):{\bar{p}}_{i}\right)$, (i=1,⋯,N);3:Calculate $K_{i}=T_{i}^{T}\left[\begin{array}{c} K_{id}\\ 0_{\left(n-v_{i}\right)\times p_{i}} \end{array}\right]$, (i∈N);4:Construct distributed observer (3) to obtain ${\hat{x}}_{i}$.


## 4. Simulation

### 4.1. Example 1

Consider a linear system (1) with 4 subsystems, the parameters of the systems are

$$\begin{array}{c} A=\left[\begin{array}{cccccc} -1 & 0 & 0 & 0 & 0 & 0\\ -1 & 1 & 1 & 0 & 0 & 0\\ 1 & -2 & -1 & -1 & 1 & 1\\ 0 & 0 & 0 & -1 & 0 & 0\\ -8 & 1 & -1 & -1 & -2 & 0\\ 4 & -0.5 & 0.5 & 0 & 0 & -4 \end{array}\right],B=\left[\begin{array}{c} 1\\ 1\\ 1\\ 1\\ 1\\ 1 \end{array}\right],\\ C=\left[\begin{array}{c} \underline{\begin{array}{cccccc} 1 & 0 & 0 & 2 & 0 & 0\\ 2 & 0 & 0 & 1 & 0 & 0 \end{array}}\\ \underline{\begin{array}{cccccc} 2 & 0 & 1 & 0 & 0 & 1 \end{array}}\\ \underline{\begin{array}{cccccc} 0 & 0 & 0 & 2 & 0 & 0 \end{array}}\\ \begin{array}{cccccc} 1 & 0 & 2 & 0 & 0 & 0\\ 2 & 0 & 4 & 0 & 0 & 0 \end{array} \end{array}\right]=\left[\begin{array}{c} C_{1}\\ C_{2}\\ C_{3}\\ C_{4} \end{array}\right], \end{array}$$

**Figure 1:**
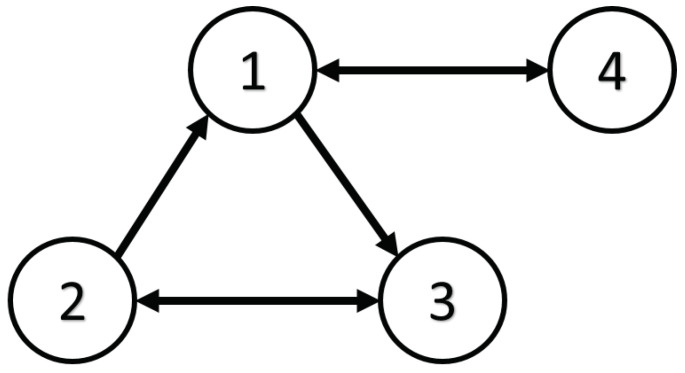
The communication graph topology.

Here, it can be seen that $\left(A,C_{i}\right)$ is neither observable nor detectable for all (i∈N) but $\left(A,C\right)$ is observable. The topology of the graph is illustrated by Figure 1; the Laplacian matrix L can be obtained.

$$\begin{array}{c} L=\left[\begin{array}{cccc} 2 & -1 & 0 & -1\\ 0 & 1 & -1 & 0\\ -1 & -1 & 2 & 0\\ -1 & 0 & 0 & 1 \end{array}\right] \end{array}$$

Furthermore, we assume that the time delay is τ=0.4 and the initial values for the target system and all observers are initialized at [0 0 0 0 0 0 ${]}^{T}$. By setting $\theta ={\left[\begin{array}{cccc} 0.25 & 0.25 & 0.25 & 0.25 \end{array}\right]}^{T}$, c=1, γ=100, the LMI (16) is feasible for $P_{o}$, $P_{u}$, $Q_{o}$, $Q_{u}$ and $X_{d}$, we can obtain:

$$\begin{array}{c} T_{1}=\left[\begin{array}{cccccc} 1 & 0 & 0 & 0 & 0 & 0\\ 0 & 0 & 0 & 1 & 0 & 0\\ 0 & 1 & 0 & 0 & 0 & 0\\ 0 & 0 & 1 & 0 & 0 & 0\\ 0 & 0 & 0 & 0 & 1 & 0\\ 0 & 0 & 0 & 0 & 0 & 1 \end{array}\right],T_{2}=\left[\begin{array}{cccccc} 0.5145 & 0 & 0 & 0 & 0 & -0.8575\\ -0.7197 & 0.5437 & 0 & 0 & 0 & -0.4318\\ 0.3597 & 0.6474 & 0.6363 & 0 & 0 & 0.2158\\ 0.1896 & 0.3413 & -0.4929 & 0.7692 & 0 & 0.1138\\ 0.041 & 0.0738 & -0.1066 & -0.1148 & 0.9837 & 0.0246\\ 0.2245 & 0.4041 & -0.5837 & -0.6286 & -0.1796 & 0.1347 \end{array}\right],\\ T_{3}=\left[\begin{array}{cccccc} 0 & 0 & 0 & 1 & 0 & 0\\ 1 & 0 & 0 & 0 & 0 & 0\\ 0 & 1 & 0 & 0 & 0 & 0\\ 0 & 0 & 1 & 0 & 0 & 0\\ 0 & 0 & 0 & 0 & 1 & 0\\ 0 & 0 & 0 & 0 & 0 & 1 \end{array}\right],T_{4}=\left[\begin{array}{cccccc} 0.7474 & 0 & 0 & 0 & 0 & -0.6644\\ -0.2963 & 0.8950 & 0 & 0 & 0 & -0.3333\\ 0.1695 & 0.1271 & 0.9585 & 0 & 0 & 0.1906\\ 0.4946 & 0.3709 & -0.2473 & 0.4971 & 0 & 0.5564\\ 0.2047 & 0.1535 & -0.1023 & -0.6268 & 0.6915 & 0.2303\\ 0.1959 & 0.1469 & -0.0980 & -0.6000 & -0.7224 & 0.2204 \end{array}\right], \end{array}$$

$$\begin{array}{c} P_{o}=\left[\begin{array}{ccccccccccccc} 15.3517 & -3.3894 & 0 & 0 & 0 & 0 & 0 & 0 & 0 & 0 & 0 & 0 & 0\\ -3.3894 & 5.7454 & 0 & 0 & 0 & 0 & 0 & 0 & 0 & 0 & 0 & 0 & 0\\ 0 & 0 & 14.0058 & -11.4503 & 6.2228 & 16.8137 & -4.9765 & 0 & 0 & 0 & 0 & 0 & 0\\ 0 & 0 & -11.4503 & 14.3667 & -13.2043 & -16.2162 & 2.1165 & 0 & 0 & 0 & 0 & 0 & 0\\ 0 & 0 & 6.2228 & -13.2043 & 37.6463 & 2.3724 & 5.1665 & 0 & 0 & 0 & 0 & 0 & 0\\ 0 & 0 & 16.8137 & -16.2162 & 2.3724 & 26.9624 & -8.4827 & 0 & 0 & 0 & 0 & 0 & 0\\ 0 & 0 & -4.9765 & 2.1165 & 5.1665 & -8.4827 & 5.6695 & 0 & 0 & 0 & 0 & 0 & 0\\ 0 & 0 & 0 & 0 & 0 & 0 & 0 & 7.4470 & 0 & 0 & 0 & 0 & 0\\ 0 & 0 & 0 & 0 & 0 & 0 & 0 & 0 & 17.8167 & -2.0586 & 4.6038 & 15.8194 & -4.2174\\ 0 & 0 & 0 & 0 & 0 & 0 & 0 & 0 & -2.0586 & 2.0797 & 2.4235 & -3.0427 & 0.1155\\ 0 & 0 & 0 & 0 & 0 & 0 & 0 & 0 & 4.6038 & 2.4235 & 20.7443 & 3.0464 & 6.2001\\ 0 & 0 & 0 & 0 & 0 & 0 & 0 & 0 & 15.8194 & -3.0427 & 3.0464 & 17.4158 & -4.3726\\ 0 & 0 & 0 & 0 & 0 & 0 & 0 & 0 & -4.2174 & 0.1155 & 6.2001 & -4.3726 & 6.6632 \end{array}\right], \end{array}$$

$$\begin{array}{c} P_{u}=\left[\begin{array}{ccccccccccc} 3.5623 & 2.2813 & 0.5341 & 0.5545 & 0 & 0 & 0 & 0 & 0 & 0 & 0\\ 2.2813 & 2.9214 & 1.5320 & 0.5091 & 0 & 0 & 0 & 0 & 0 & 0 & 0\\ 0.5341 & 1.5320 & 1.6951 & 1.0928 & 0 & 0 & 0 & 0 & 0 & 0 & 0\\ 0.5545 & 0.5091 & 1.0928 & 3.6807 & 0 & 0 & 0 & 0 & 0 & 0 & 0\\ 0 & 0 & 0 & 0 & 0.3745 & 0 & 0 & 0 & 0 & 0 & 0\\ 0 & 0 & 0 & 0 & 0 & 13.0474 & -1.6092 & -1.4060 & -0.8643 & -0.8049 & 0\\ 0 & 0 & 0 & 0 & 0 & -1.6092 & 2.2418 & 1.2909 & 0.1959 & 0.2437 & 0\\ 0 & 0 & 0 & 0 & 0 & -1.4060 & 1.2909 & 1.3807 & 0.5249 & 0.3094 & 0\\ 0 & 0 & 0 & 0 & 0 & -0.8643 & 0.1959 & 0.5249 & 0.7437 & 0.9670 & 0\\ 0 & 0 & 0 & 0 & 0 & -0.8049 & 0.2437 & 0.3094 & 0.9670 & 3.3237 & 0\\ 0 & 0 & 0 & 0 & 0 & 0 & 0 & 0 & 0 & 0 & 0.4115 \end{array}\right], \end{array}$$

$$\begin{array}{c} Q_{o}=\left[\begin{array}{ccccccccccccc} 34.8182 & -0.4649 & 0 & 0 & 0 & 0 & 0 & 0 & 0 & 0 & 0 & 0 & 0\\ -0.4649 & 3.4110 & 0 & 0 & 0 & 0 & 0 & 0 & 0 & 0 & 0 & 0 & 0\\ 0 & 0 & 10.5226 & -12.1906 & -3.5589 & 2.7984 & -3.0916 & 0 & 0 & 0 & 0 & 0 & 0\\ 0 & 0 & -12.1906 & 25.5771 & 9.9243 & -2.4880 & 4.2512 & 0 & 0 & 0 & 0 & 0 & 0\\ 0 & 0 & -3.5589 & 9.9243 & 16.4028 & 0.2881 & 2.5395 & 0 & 0 & 0 & 0 & 0 & 0\\ 0 & 0 & 2.7984 & -2.4880 & 0.2881 & 3.2461 & -0.0809 & 0 & 0 & 0 & 0 & 0 & 0\\ 0 & 0 & -3.0916 & 4.2512 & 2.5395 & -0.0809 & 3.4821 & 0 & 0 & 0 & 0 & 0 & 0\\ 0 & 0 & 0 & 0 & 0 & 0 & 0 & 3.9413 & 0 & 0 & 0 & 0 & 0\\ 0 & 0 & 0 & 0 & 0 & 0 & 0 & 0 & 3.8071 & -0.8010 & -1.0720 & -0.9268 & -0.6791\\ 0 & 0 & 0 & 0 & 0 & 0 & 0 & 0 & -0.8010 & 12.0017 & 6.8615 & 3.0779 & 2.2601\\ 0 & 0 & 0 & 0 & 0 & 0 & 0 & 0 & -1.0720 & 6.8615 & 11.2034 & 2.2925 & 3.5806\\ 0 & 0 & 0 & 0 & 0 & 0 & 0 & 0 & -0.9268 & 3.0779 & 2.2925 & 3.0897 & 1.9743\\ 0 & 0 & 0 & 0 & 0 & 0 & 0 & 0 & -0.6791 & 2.2601 & 3.5806 & 1.9743 & 3.2610 \end{array}\right], \end{array}$$

$$\begin{array}{c} Q_{u}=\left[\begin{array}{ccccccccccc} 1.2020 & 0.8227 & -0.2075 & 0.2855 & 0 & 0 & 0 & 0 & 0 & 0 & 0\\ 0.8227 & 1.1699 & 0.2158 & -0.0638 & 0 & 0 & 0 & 0 & 0 & 0 & 0\\ -0.2075 & 0.2158 & 0.6940 & 0.6527 & 0 & 0 & 0 & 0 & 0 & 0 & 0\\ 0.2855 & -0.0638 & 0.6527 & 3.8573 & 0 & 0 & 0 & 0 & 0 & 0 & 0\\ 0 & 0 & 0 & 0 & 0.1902 & 0 & 0 & 0 & 0 & 0 & 0\\ 0 & 0 & 0 & 0 & 0 & 5.2207 & -1.1126 & -0.5123 & 0.4465 & -0.6597 & 0\\ 0 & 0 & 0 & 0 & 0 & -1.1126 & 1.1477 & 0.6517 & -0.3007 & 0.1264 & 0\\ 0 & 0 & 0 & 0 & 0 & -0.5123 & 0.6517 & 0.7548 & 0.0717 & 0.1230 & 0\\ 0 & 0 & 0 & 0 & 0 & 0.4465 & -0.3007 & 0.0717 & 0.6905 & 0.9133 & 0\\ 0 & 0 & 0 & 0 & 0 & -0.6597 & 0.1264 & 0.1230 & 0.9133 & 3.6940 & 0\\ 0 & 0 & 0 & 0 & 0 & 0 & 0 & 0 & 0 & 0 & 0.1530 \end{array}\right], \end{array}$$

$$\begin{array}{c} X_{d}=\left[\begin{array}{cccccc} -10.6407 & -27.4692 & 0 & 0 & 0 & 0\\ -6.1193 & 0.0343 & 0 & 0 & 0 & 0\\ 0 & 0 & -0.0775 & 0 & 0 & 0\\ 0 & 0 & 8.0686 & 0 & 0 & 0\\ 0 & 0 & 7.0968 & 0 & 0 & 0\\ 0 & 0 & 4.5127 & 0 & 0 & 0\\ 0 & 0 & 6.8583 & 0 & 0 & 0\\ 0 & 0 & 0 & -12.7959 & 0 & 0\\ 0 & 0 & 0 & 0 & 0.0148 & 0.0295\\ 0 & 0 & 0 & 0 & 1.7084 & 3.4168\\ 0 & 0 & 0 & 0 & -0.7082 & -1.4164\\ 0 & 0 & 0 & 0 & 1.2386 & 2.4773\\ 0 & 0 & 0 & 0 & -0.1463 & -0.2927 \end{array}\right], \end{array}$$

By Algorithm 1, we can obtain $K_{d}=P_{o}^{-1}X_{d}$, $K_{id}=K_{d}\left(\left({\bar{v}}_{i-1}+1\right):{\bar{v}}_{i},\left({\bar{p}}_{i-1}+1\right):{\bar{p}}_{i}\right)$, (i=1,⋯,N), and then calculate the observer gain matrix and $K_{i}=T_{i}^{T}\left[\begin{array}{c} K_{id}\\ 0_{\left(n-v_{i}\right)\times p_{i}} \end{array}\right]$, (i∈N):

$$\begin{array}{c} K_{1}=\left[\begin{array}{cc} -1.0673 & -2.0558\\ 0 & 0\\ 0 & 0\\ -1.6947 & -1.2068\\ 0 & 0\\ 0 & 0 \end{array}\right],K_{2}=\left[\begin{array}{c} -26.38042013\\ 56.63379691\\ -17.29984844\\ 29.67078404\\ 34.17659073\\ -16.86259616 \end{array}\right],K_{3}=\left[\begin{array}{c} 0\\ 0\\ 0\\ -1.7183\\ 0\\ 0 \end{array}\right],K_{4}=\left[\begin{array}{cc} -0.62123138 & -1.24249720\\ 10.49287963 & 20.98590120\\ -4.58478301 & -9.16960098\\ -1.35814064 & -2.71629425\\ 3.59918835 & 7.19844585\\ -0.38187038 & -0.76362898 \end{array}\right], \end{array}$$

Finally, we can calculate out that:

${\bar{A}}_{1}=\left[\begin{array}{cccccc} -6.1789 & -4.1904 & 0 & 0 & 0 & 0\\ -4.1083 & -5.5962 & 0 & 0 & 0 & 0\\ -1.0000 & 0 & 1.0000 & 1.0000 & 0 & 0\\ 1.0000 & -1.0000 & -2.0000 & -1.0000 & 1.0000 & 1.0000\\ -8.0000 & -1.0000 & 1.0000 & -1.0000 & -2.0000 & 0\\ 4.0000 & 0 & -0.5000 & 0.5000 & 0 & -4.0000 \end{array}\right],$

${\bar{A}}_{2}=\left[\begin{array}{cccccc} -4.8181 & -0.0731 & 0.7236 & 0 & 0 & 0\\ 7.5065 & -105.9808 & 90.1845 & 0 & 0 & 0\\ 2.5967 & -24.0989 & 19.4192 & -0.7308 & 0.6469 & 0\\ 8.0248 & -80.3678 & 69.7473 & -0.4339 & -0.5011 & 0\\ 2.0218 & -58.4826 & 51.9907 & -1.3057 & -2.1083 & 0\\ 1.3553 & 0.6556 & 1.6756 & 0.9311 & -0.4108 & -1.0000 \end{array}\right],$

${\bar{A}}_{3}=\left[\begin{array}{cccccc} -4.4366 & 0 & 0 & 0 & 0 & 0\\ 0 & -1.0000 & 0 & 0 & 0 & 0\\ 0 & -1.0000 & 1.0000 & 1.0000 & 0 & 0\\ -1.0000 & 1.0000 & -2.0000 & -1.0000 & 1.0000 & 1.0000\\ -1.0000 & -8.0000 & 1.0000 & -1.0000 & -2.0000 & 0\\ 0 & 4.0000 & -0.5000 & 0.5000 & 0 & -4.0000 \end{array}\right],$

${\bar{A}}_{4}=\left[\begin{array}{cccccc} -5.0960 & 0.7318 & -2.5400 & 0 & 0 & 0\\ 33.9331 & -13.2978 & 101.9961 & 0 & 0 & 0\\ -11.1711 & 2.6244 & -34.4621 & -0.1802 & 1.3863 & 0\\ 16.7904 & -4.6552 & 40.6170 & -0.9535 & -0.3576 & 0\\ 16.3406 & -5.0310 & 53.0587 & -2.6328 & -2.1480 & 0\\ 5.2998 & -1.9548 & 1.8379 & 2.7892 & 0.9031 & -1.0000 \end{array}\right],$

$F_{1}=\left[\begin{array}{cccccc} 0 & 0 & 0 & 0 & 0 & 0\\ 0 & 0 & 0 & 0 & 0 & 0\\ 0 & 1.0000 & 0 & 0 & 0 & 0\\ 0 & 0 & 1.0000 & 0 & 0 & 0\\ 0 & 0 & 0 & 0 & 1.0000 & 0\\ 0 & 0 & 0 & 0 & 0 & 1.0000 \end{array}\right],$

$F_{2}=\left[\begin{array}{cccccc} 0 & 0 & 0 & 0 & 0 & 0\\ -6.04803\times 10^{-6} & -1.08865\times 10^{-5} & 1.57249\times 10^{-5} & 1.69345\times 10^{-5} & 4.83842\times 10^{-6} & -3.62882\times 10^{-6}\\ 6.04803\times 10^{-6} & 1.08865\times 10^{-5} & -1.57249\times 10^{-5} & -1.69345\times 10^{-5} & -4.83842\times 10^{-6} & 3.62882\times 10^{-6}\\ -1.28517\times 10^{-17} & -2.31331\times 10^{-17} & 3.34144\times 10^{-17} & 3.59848\times 10^{-17} & 1.02814\times 10^{-17} & -7.71102\times 10^{-18}\\ 1.20961\times 10^{-5} & 2.17729\times 10^{-5} & -3.14498\times 10^{-5} & -3.38690\times 10^{-5} & -9.67685\times 10^{-6} & 7.25764\times 10^{-6}\\ 0.22448675 & 0.40407614 & -0.58366555 & -0.62856289 & -0.17958940 & 0.13469205 \end{array}\right],$

$F_{3}=\left[\begin{array}{cccccc} 0 & 0 & 0 & 0 & 0 & 0\\ 1.0000 & 0 & 0 & 0 & 0 & 0\\ 0 & 1.0000 & 0 & 0 & 0 & 0\\ 0 & 0 & 1.0000 & 0 & 0 & 0\\ 0 & 0 & 0 & 0 & 1.0000 & 0\\ 0 & 0 & 0 & 0 & 0 & 1.0000 \end{array}\right],$

**Figure 2:**
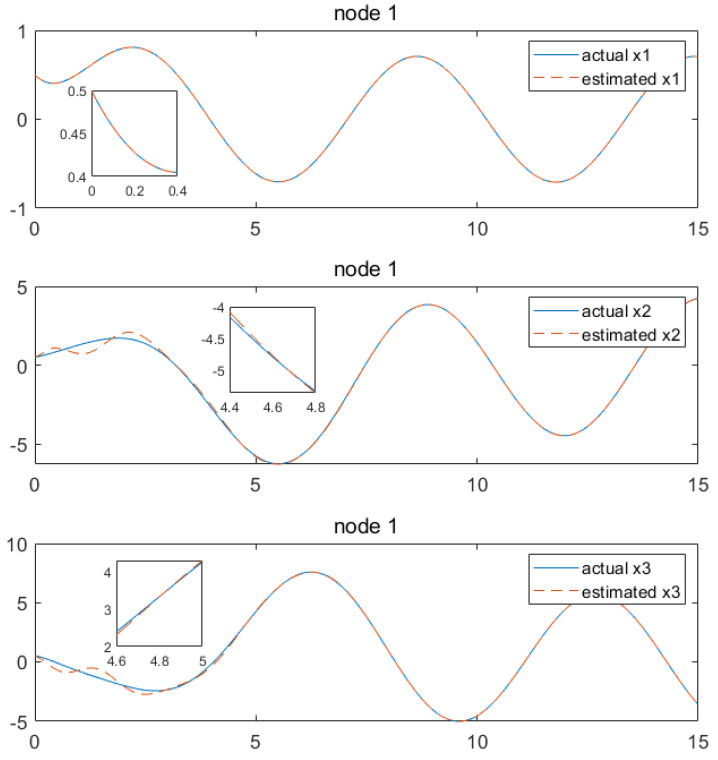
State estimations of $x_{1}-x_{3}$ by node 1.

**Figure 3:**
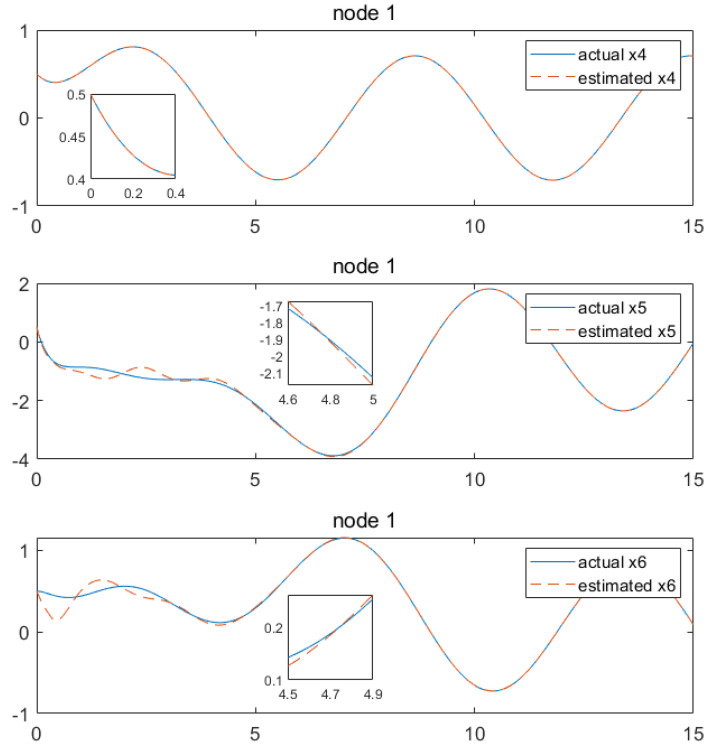
State estimations of $x_{4}-x_{6}$ by node 1.

**Figure 4:**
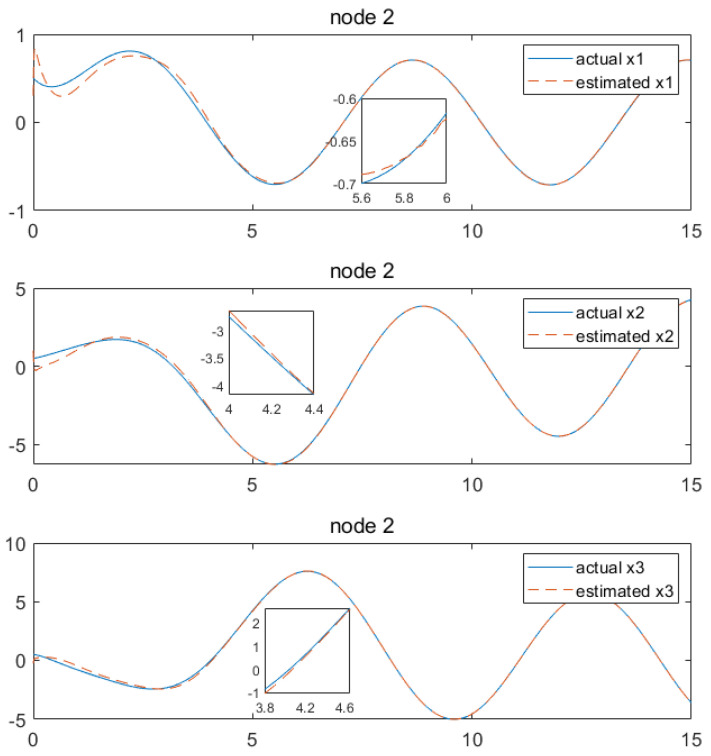
State estimations of $x_{1}-x_{3}$ by node2.

**Figure 5:**
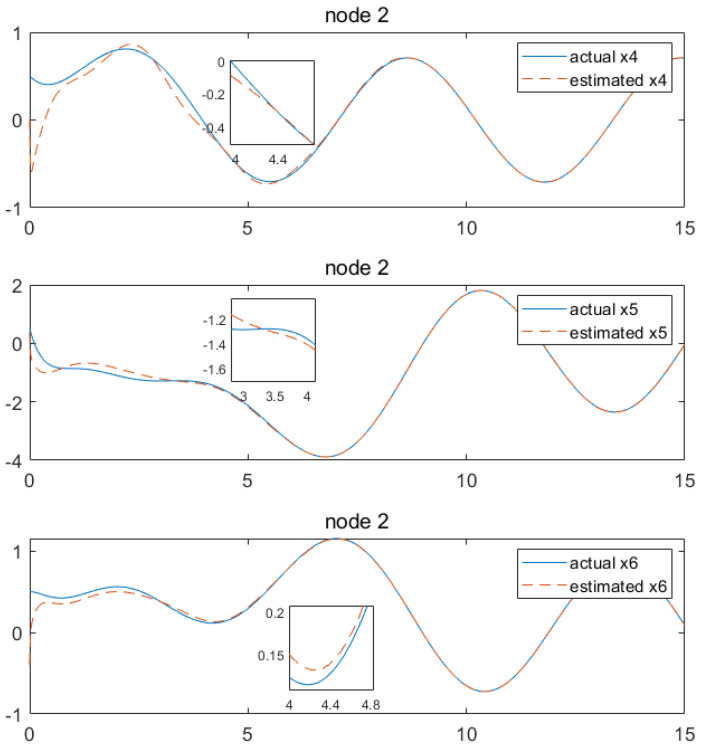
State estimations of $x_{4}-x_{6}$ by node 2.

**Figure 6:**
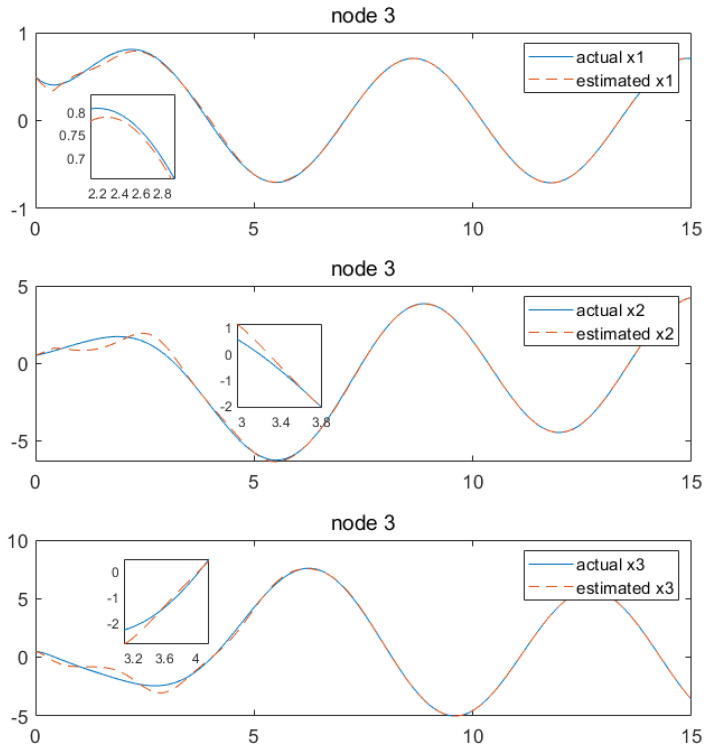
State estimations of $x_{1}-x_{3}$ by node 3.

**Figure 7:**
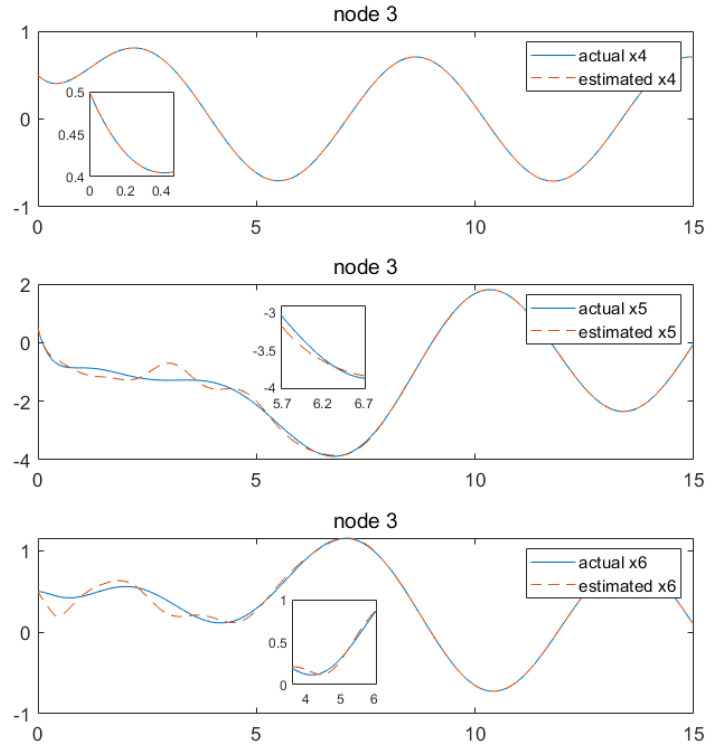
State estimations of $x_{4}-x_{6}$ by node 3.

**Figure 8:**
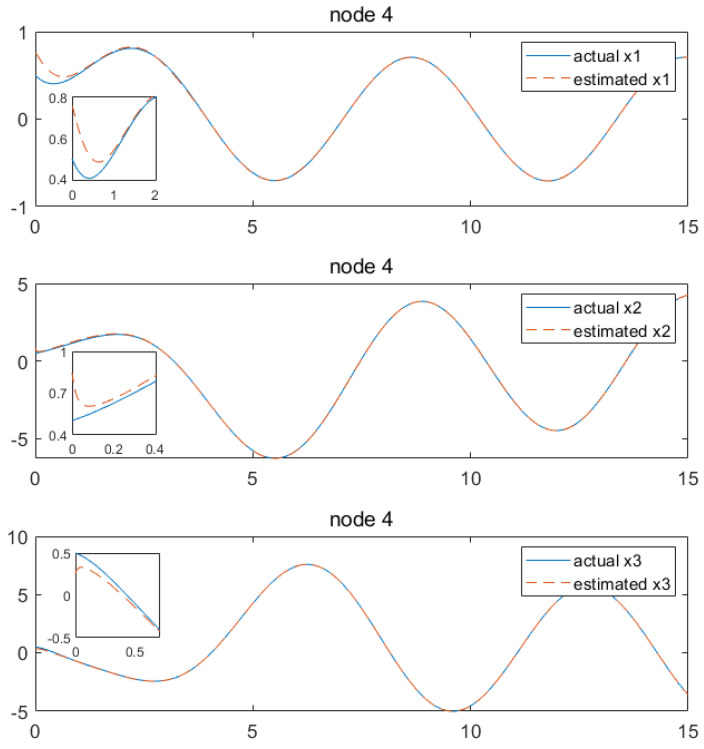
State estimations of $x_{1}-x_{3}$ by node 4.

$F_{4}=\left[\begin{array}{cccccc} -3.54579\times 10^{-6} & -2.65889\times 10^{-6} & 1.77380\times 10^{-6} & 1.08600\times 10^{-5} & 1.30754\times 10^{-5} & -3.98924\times 10^{-6}\\ -5.67914\times 10^{-6} & -4.25863\times 10^{-6} & 2.84102\times 10^{-6} & 1.73940\times 10^{-5} & 2.09424\times 10^{-5} & -6.38940\times 10^{-6}\\ -9.54425\times 10^{-6} & -7.15697\times 10^{-6} & 4.77456\times 10^{-6} & 2.92320\times 10^{-5} & 3.51953\times 10^{-5} & -1.07379\times 10^{-5}\\ -3.26957\times 10^{-6} & -2.45176\times 10^{-6} & 1.63562\times 10^{-6} & 1.00140\times 10^{-5} & 1.20569\times 10^{-5} & -3.67848\times 10^{-6}\\ -5.13258\times 10^{-6} & -3.84878\times 10^{-6} & 2.56760\times 10^{-6} & 1.57200\times 10^{-5} & 1.89269\times 10^{-5} & -5.77448\times 10^{-6}\\ 0.195899675 & 0.146899756 & -0.097999837 & -0.599999004 & -0.722398801 & 0.220399634 \end{array}\right]$ Based on the above calculation results, the distributed observer (4) can be constructed. The state and their estimates of each node are depicted in Figure 2, Figure 3, Figure 4, Figure 5, Figure 6, Figure 7 and Figure 8. It can be seen that all estimates converge to the actual state asymptotically.

In Figure 2 and Figure 3 for node 1, the state estimation $x_{1}$ and $x_{4}$ reach the convergence at the beginning, while $x_{2}$, $x_{3}$, $x_{5}$ and $x_{6}$ achieve convergence later, at around 4.7 s.

In Figure 4 and Figure 5 for node 2, the state estimations of $x_{1}$ and $x_{4}$ can be realized at approximately 5.9 s, while $x_{2}$, $x_{6}$, $x_{3}$ and $x_{5}$ achieve convergence at around 3.4 s, 4.4 s, 4.7 s, 4.2 s and 3.4 s, respectively.

Figure 6 and Figure 7 provide the state estimations by node 3, which show that the state estimation $x_{1}$ has the convergence at approximately 2.6 s, while $x_{2}$ and $x_{3}$ achieve convergence at around 3.6 s. Lastly, $x_{5}$ and $x_{6}$ convergence can be achieved at 6.4 and 4.5 s, respectively.

**Figure 9:**
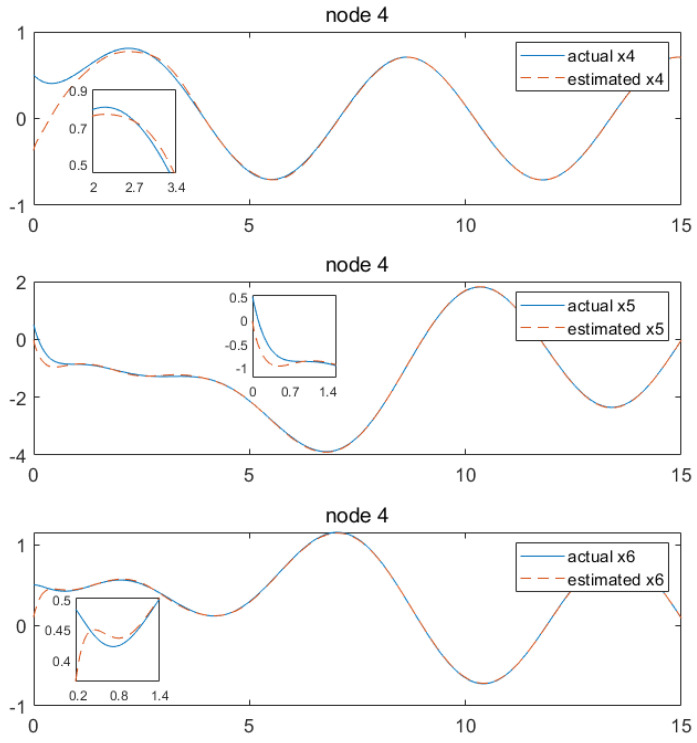
State estimations of $x_{4}-x_{6}$ by node 4.

**Figure 10:**
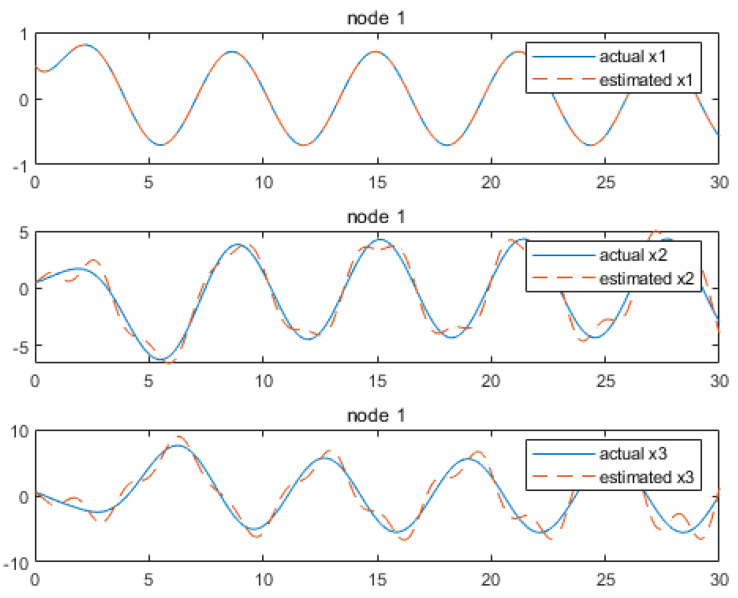
State estimations of $x_{1}-x_{3}$ by node 1 (Latency set to 0.6 s).

**Figure 11:**
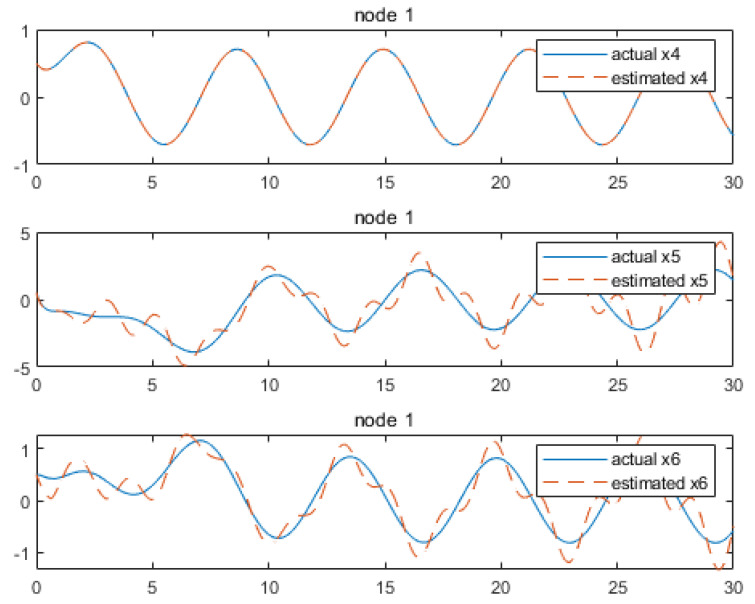
State estimations of $x_{4}-x_{6}$ by node 1 (Latency set to 0.6 s).

In Figure 8 and Figure 9 for node 4, the convergences of the state estimations of $x_{1}$, $x_{2}$, $x_{3}$, $x_{4}$, $x_{5}$ and $x_{6}$ can be realized at 1 s, 0.2 s, 0.4 s, 2.7 s, 0.9 s and 1.1 s, respectively. In the depicted scenario, each node observer exhibits rapid convergence. That is, we conclude that the convergence characteristics depend greatly on the output information received by each node, showing the distributed characteristics of the observer. Currently, our method is capable of handling estimations for information communication with a time delay which is less than 0.5 s. Beyond this threshold, convergence cannot be achieved. As illustrated, when the time delay is set as 0.6 s, taking node 1 as an example, it can be observed from Figure 10 and Figure 11 that the state estimations cannot be accomplished asymptotically. This is an aspect that requires further investigation.

### 4.2. Example 2

In this part, the proposed distributed observer design strategy is tested by a mobile robot system with four sensor groups, where the information communication topology is depicted in Figure 1. The robot dynamic system is formulated as [33]

$$\left\{\begin{array}{c} \dot{\bar{x}}=\bar{v}\cos\chi \\ \dot{\bar{y}}=\bar{v}\sin\chi \\ \dot{\chi }=\zeta \end{array}\right.$$

where $\left(\;\bar{x},\;\bar{y}\right)$ denotes the center position of the robot, χ indicates the heading angle in the inertial frame, and $\;\bar{v}$ and ζ are, respectively, the linear velocity and angular velocity. Define $x=\mathrm{col}\left\{\bar{x},{\bar{v}}^{x},\bar{y},{\bar{v}}^{y}\right\}$ and $u=\mathrm{col}\{{\bar{u}}^{x},{\bar{u}}^{y}\}$, where ${\bar{v}}^{x}$ and ${\bar{v}}^{y}$ are the components of the linear velocities along the X- and Y-axes, respectively, and ${\bar{u}}^{x}$ and ${\bar{u}}^{y}$ are the components of the input signals along the X- and Y-axes, respectively. The system model can be linearized and the system matrices are:

$$\begin{array}{c} A=I_{2}⊗\left[\begin{array}{cc} 0 & 1\\ 0 & 0 \end{array}\right],B=I_{2}⊗\left[\begin{array}{c} 0\\ 1 \end{array}\right],C=\left[\begin{array}{c} \underline{\begin{array}{cccc} 1 & 0 & 0 & 0 \end{array}}\\ \underline{\begin{array}{cccc} 0 & 1 & 0 & 0 \end{array}}\\ \underline{\begin{array}{cccc} 0 & 0 & 1 & 0 \end{array}}\\ \begin{array}{cccc} 0 & 0 & 0 & 1 \end{array} \end{array}\right]=\left[\begin{array}{c} C_{1}\\ C_{2}\\ C_{3}\\ C_{4} \end{array}\right] \end{array}$$

For this configuration of sensors, we obtain that $rank(O_{1})=2$, $rank(O_{2})=1$, $rank(O_{2})=2$, and $rank(O_{4})=1$. Furthermore, we confirm that (C,A) is observable. Assuming a delay of 0.4, with the target system initialized at a value of [0.7 0.7 0.7 0.7 ${]}^{T}$, and the observers labeled 1 through 4 are initialized at values of [0.3 0.3 0.3 0.3 ${]}^{T}$, [ 0.2 0.2 0.2 0.2 ${]}^{T}$, [ 0 0 0 0${]}^{T}$ and [0.9 0.9 0.9 0.9 ${]}^{T}$, respectively, and with $\theta ={\left[\begin{array}{cccc} 0.25 & 0.25 & 0.25 & 0.25 \end{array}\right]}^{T}$, c=1, γ=100, the LMI (16) is feasible for $P_{o}$, $P_{u}$, $Q_{o}$, $Q_{u}$ and $X_{d}$, we can obtain:

$$\begin{array}{c} P_{o}=\left[\begin{array}{cccccc} 8.96 & -9.9626 & 0 & 0 & 0 & 0\\ -9.9626 & 12.6876 & 0 & 0 & 0 & 0\\ 0 & 0 & 4.4167 & 0 & 0 & 0\\ 0 & 0 & 0 & 24.7045 & -29.4648 & 0\\ 0 & 0 & 0 & -29.4648 & 35.7729 & 0\\ 0 & 0 & 0 & 0 & 0 & 4.4167 \end{array}\right], \end{array}$$

$$\begin{array}{c} Q_{o}=\left[\begin{array}{cccccc} 7.214 & 1.8942 & 0 & 0 & 0 & 0\\ 1.8942 & 18.4471 & 0 & 0 & 0 & 0\\ 0 & 0 & 13.6074 & 0 & 0 & 0\\ 0 & 0 & 0 & 7.6471 & 5.1647 & 0\\ 0 & 0 & 0 & 5.1647 & 57.9018 & 0\\ 0 & 0 & 0 & 0 & 0 & 27.9153 \end{array}\right], \end{array}$$

$$\begin{array}{c} P_{u}=\left[\begin{array}{cccccccccc} 0.0736 & -0.2489 & 0 & 0 & 0 & 0 & 0 & 0 & 0 & 0\\ -0.2489 & 7.3412 & 0 & 0 & 0 & 0 & 0 & 0 & 0 & 0\\ 0 & 0 & 0.1103 & 0 & 0 & 0 & 0 & 0 & 0 & 0\\ 0 & 0 & 0 & 0.2436 & -0.6993 & 0 & 0 & 0 & 0 & 0\\ 0 & 0 & 0 & -0.6993 & 13.362 & 0 & 0 & 0 & 0 & 0\\ 0 & 0 & 0 & 0 & 0 & 0.0971 & -0.1606 & 0 & 0 & 0\\ 0 & 0 & 0 & 0 & 0 & -0.1606 & 2.834 & 0 & 0 & 0\\ 0 & 0 & 0 & 0 & 0 & 0 & 0 & 0.0869 & -0.318 & 0\\ 0 & 0 & 0 & 0 & 0 & 0 & 0 & -0.318 & 3.4807 & 0\\ 0 & 0 & 0 & 0 & 0 & 0 & 0 & 0 & 0 & 0.0813 \end{array}\right], \end{array}$$

$$\begin{array}{c} Q_{u}=\left[\begin{array}{cccccccccc} 0.0103 & -0.0879 & 0 & 0 & 0 & 0 & 0 & 0 & 0 & 0\\ -0.0879 & 2.1239 & 0 & 0 & 0 & 0 & 0 & 0 & 0 & 0\\ 0 & 0 & 0.0057 & 0 & 0 & 0 & 0 & 0 & 0 & 0\\ 0 & 0 & 0 & 0.027 & -0.1552 & 0 & 0 & 0 & 0 & 0\\ 0 & 0 & 0 & -0.1552 & 1.7186 & 0 & 0 & 0 & 0 & 0\\ 0 & 0 & 0 & 0 & 0 & 0.0164 & -0.0722 & 0 & 0 & 0\\ 0 & 0 & 0 & 0 & 0 & -0.0722 & 0.8782 & 0 & 0 & 0\\ 0 & 0 & 0 & 0 & 0 & 0 & 0 & 0.0171 & -0.081 & 0\\ 0 & 0 & 0 & 0 & 0 & 0 & 0 & -0.081 & 0.6731 & 0\\ 0 & 0 & 0 & 0 & 0 & 0 & 0 & 0 & 0 & 0.0041 \end{array}\right], \end{array}$$

$$\begin{array}{c} K_{d}=\left[\begin{array}{cccc} -12.7454 & 0 & 0 & 0\\ -10.7257 & 0 & 0 & 0\\ 0 & -2.8523 & 0 & 0\\ 0 & 0 & -73.4318 & 0\\ 0 & 0 & -61.2423 & 0\\ 0 & 0 & 0 & -5.8443 \end{array}\right], \end{array}$$

**Figure 12:**
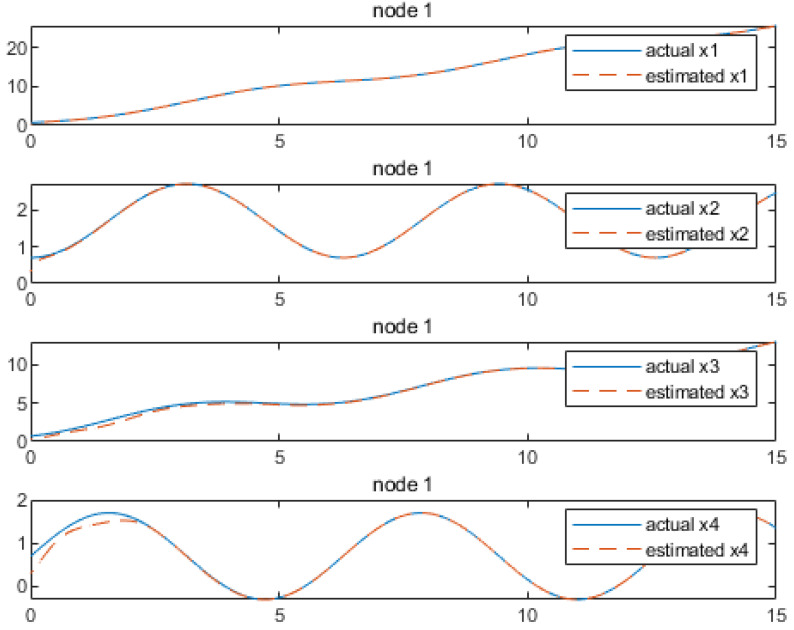
State estimations by node 1.

**Figure 13:**
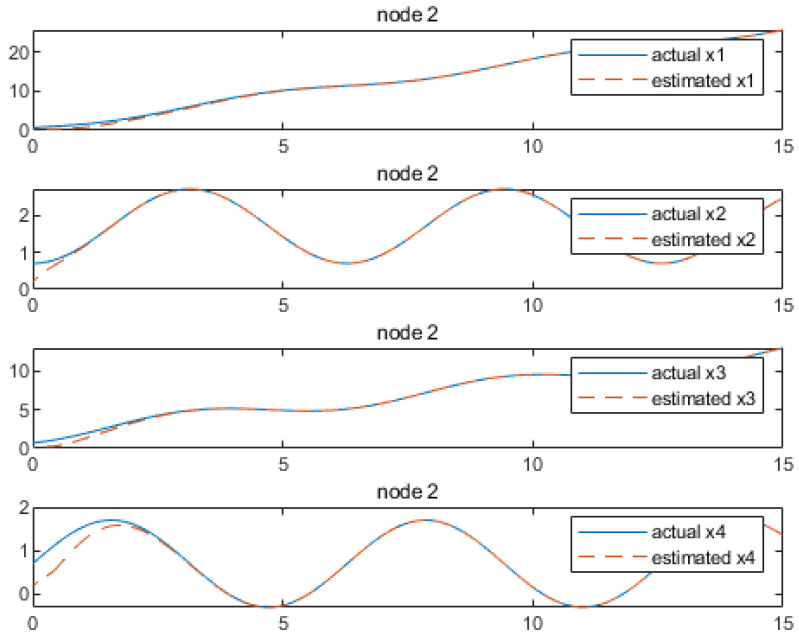
State estimations by node 2.

**Figure 14:**
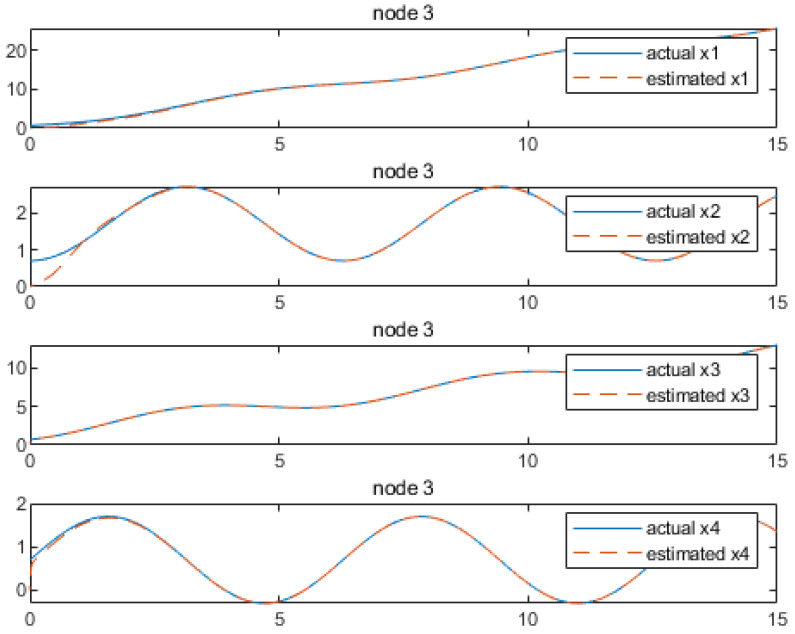
State estimations by node 3.

**Figure 15:**
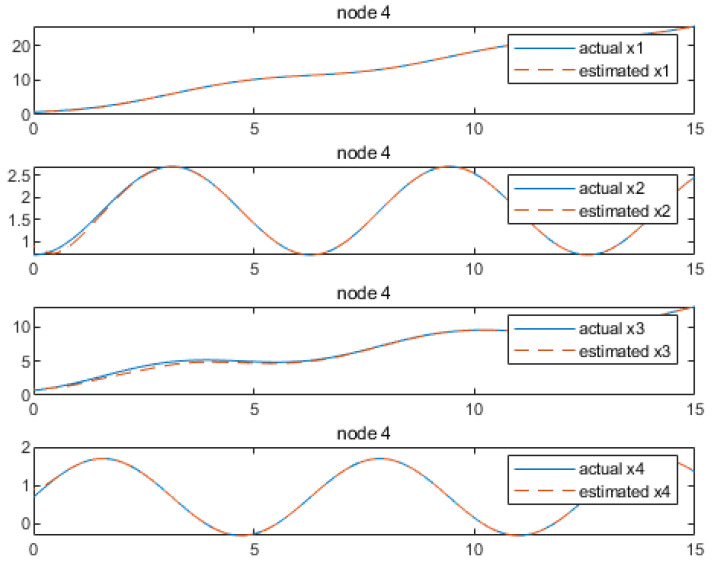
State estimations by node 4.

As a supplementary example, further elaboration on the remaining parameters is omitted; the Figure 12, Figure 13, Figure 14 and Figure 15 demonstrate that the distributed observer proposed in this study effectively estimates the states of the target system. It can be seen that all states estimates converge to the actual states asymptotically.

## 5. Conclusions

This paper addresses time-delay issues due to the information exchange in distributed observer design. Distributed observers are constructed for systems based on the information exchange among the nodes and the information transmission may lead to time delay. To deal with the time delay, for each node, an equivalent system is set up which is a model containing time-delay statistics received from its neighbours. To eliminate the negative influence of the time delay, a special Lyapunov function is constructed which contains two parts and the two parts are associated with each other. The stability analysis is carried out based on the Lyapunov function and the observer gain matrix for each distributed observer is calculated by an LMI. How to deal with communication time delays which are not only time varying but also unknown will be one of our further focuses of research. Furthermore, considering a more complicated model with parameter variations or unknown inputs is also an interesting issue that deserves to be investigated in the future.

## Data Availability

No new data were created.

## Abbreviations

The following abbreviations are used in this manuscript:

LTI	Linear Time-Invariant
LMI	Linear Matrix Inequality
UIO	Unknown Input Observer
